# Innovative Development of Batch Dyed 3D Printed Acrylonitrile/Butadiene/Styrene Objects

**DOI:** 10.3390/molecules26216637

**Published:** 2021-11-02

**Authors:** Suzana Kutnjak-Mravlinčić, Ana Sutlović, Martinia Ira Glogar, Sanja Ercegović Ražić, Damir Godec

**Affiliations:** 1 Design and Production of Footwear, Faculty of Textile Technology, University of Zagreb, 42000 Varaždin, Croatia; skutnjak@ttf.unizg.hr; 2 Department of Textile Chemistry and Ecology, Faculty of Textile Technology, University of Zagreb, 10000 Zagreb, Croatia; martinia.glogar@ttf.hr; 3 Department of Materials, Fibres and Textile Testing, Faculty of Textile Technology, University of Zagreb, 10000 Zagreb, Croatia; sanja.ercegovic@ttf.unizg.hr; 4 Department of Technology, Faculty of Mechanical Engineering and Naval Architecture, University of Zagreb, 10000 Zagreb, Croatia; damir.godec@fsb.hr

**Keywords:** additive manufacturing, 3D printing, acrylonitrile/butadiene/styrene polymer, batch post dyeing, disperse dyes, CIELab system, abrasion resistance, footwear design

## Abstract

According to the great impact of additive technology on the development of modern industry, a lot of research is being done to obtain 3D printed parts with better properties. This research is extremely important because there are no scientific papers in the field of post dyeing of acrylonitrile/butadiene/styrene (ABS) 3D printed parts. The experiment was carried out using disperse dyes on ABS specimens. The obtained coloration of the specimens was in the primary colors (yellow, red, and blue) in the specified dyestuff concentration range and was evaluated using an objective CIELab system. Based on the obtained color parameters, remission values and Kubelka-Munk coefficient, dye mixtures and an ombre effect were performed to obtain patterns in the desired hues. Abrasion resistance of disperse dyed specimens was tested using different abrasive materials over a wide range of fineness to simulate different indoor and outdoor soils and was compared to abrasion resistance of specimens produced from the industrially dyed wire with the master batch. The results show that 3D printed ABS products can be produced in one or more desired shades with satisfactory abrasion resistance. This undoubtedly represents the added value of 3D printed ABS parts and extends their application to the field of creative industries and design, specifically footwear design.

## 1. Introduction

**Figure 1:**
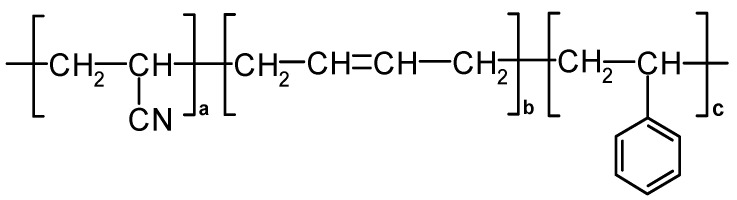
Chemical structure of acrylonitrile/butadiene/styrene a—polyacrylonitrile; b—polybutadiene; c—polystyrene [17].

One of the Additive Manufacturing (AM) processes that enables rapid prototyping or small batch production is the Fused Deposition Modeling (FDM) process. Additive manufacturing based on FDM or the synonymous 3D printing has already had a significant impact on various industries and will continue to be a key technology in the coming years as the technology evolves [1,2,3,4,5,6]. 3D printing is a powerful technology that is leading to significant changes in many areas, including the fashion industry. Design and 3D printing have become increasingly popular in the manufacture of garments and footwear. However, it is more commonly used in the production of small products such as accessories, jewelry, shoe heels, etc. [7,8,9,10,11,12]. Although, there are a number of different materials that can be used in the FDM process, acrylonitrile/butadiene/styrene (ABS) is one of the most commonly used materials for the fabrication of a variety of 3D printed objects for different applications due to its dimensional stability and low glass transition temperature [4,5,6]. ABS (Figure 1) is a thermoplastic amorphous polymer prepared by the polymerization of styrene, acrylonitrile, and polybutadiene. Each of these monomers adds some advantage to the properties of ABS: acrylonitrile provides chemical and thermal stability, butadiene increases toughness and impact strength, and styrene gives the plastic a beautiful and glossy appearance [13,14,15,16,17].

One of the limitations of FDM-ABS 3D printing on low-cost desktop printers is the printing of monochrome or dual-color 3D printed objects (depending on the number of printer nozzles), which makes it difficult to meet the requirements for high-quality reproduction, and therefore possible improvements in FDM color 3D printing are being investigated [18,19,20,21]. A positive example of the possibility of color 3D printing is the patented three-dimensional color gradient print cartridge for modelling FDM polymer technology in ABS filament [8]. The possibility of obtaining colored 3D printed objects as a post-treatment process is realized, for example, by applying plastic films to 3D printed models treated as molds [20]. Polymer dyeing is usually done using disperse dyes with the master batch. This brings with it the lack of breadth in the range of shades, in addition to its numerous advantages, mainly the fastness properties [22,23,24,25].

Color is an inevitable parameter for the quality, functional-aesthetic properties and result of the product for the consumer and as such, a powerful communication tool. This scientific study is an important and essential step on the way to color 3D printed products, because from the review of scientific papers it appears that there is no example of a scientific study on the possibility of post coloring of 3D printed objects manufactured with FDM technology from ABS with disperse dyes by the process of exhaustion. The aim of this study was to evaluate colored 3D printed ABS specimens dyed in batch process with disperse dyes. The possibility of achieving colored ombre effects through the capillary movement of dyes on 3D printed ABS specimens was further investigated to obtain value-added products in the field of visual effects. According to the theory of polymer dyeing, three disperse dyes of primary hue and different chemical constitutions were used in the study. Dyeing of artificial polymeric materials with disperse dyes leads to hydrogen bonding across the -OH group of dyes [23,24]. In 3D printed materials, the diffusion of dyes into the interior of the structure is lower than for example, in synthetic fibers. However, the authors carried out preliminary studies and demonstrated that only disperse dyes are acceptable for batch dyeing of acrylonitrile/butadiene/styrene specimens. Dyes from other groups: acidic, basic, and reactive, did not show satisfactory results [10,11,12,21,22]. The abrasion resistance of post dyed 3D printed products and products made from original MakerBot ABS filament dyed with a master batch (during melt spinning) was tested. The abrasion resistance test was performed on different abrasive materials over a wide range of fineness to simulate different indoor and outdoor soils [26,27,28,29].

This research represents a contribution to the development of colored 3D printed objects because the authors do not find any scientific research in the field of post dyeing of 3D printed objects. The results are applicable in real sector and provides support for a market quick response of 3D-colored products. Additionally, the implementation of research in the field of footwear industry ensures sustainability due to the realization of the unique personalized products.

## 2. Materials and Methods

### 2.1. Acrylonitrile/Butadiene/Styrene Polymer

The polymer used for 3D printing was acrylonitrile/butadiene/styrene (ABS) filament, produced by MakerBot, MakerBot Industries, New York, NY, USA. The filament was in the form of a 1.75 mm diameter wire, industrially shaded and declared as in Natural and True Red coloration. The filament declared as Natural is actually undyed and was furtherly used for 3D printed specimens that will be subjected to a post dyeing process and compared in further analyses to the specimens printed from the True Red filament industrially dyed with the master batch.

### 2.2. Disperse Dyes

The information on the dyes used, can be found in Table 1.

### 2.3. 3D Printing

The specimens were produced using the FDM process from ABS on a MakerBot Replicator 2X desktop printer from MakerBot Industries, New York, USA. The printing parameters of the specimens are listed in Table 2.

It should be explained that in the first step, the specimens of smooth surface structure were printed and post dyeing process in dyestuff concentration gradation was performed on these specimen as shown in in Section 3.1. The smoothness of the surface also allows more accurate spectrophotometric measurements and color analysis.

Furthermore, to accentuate the ombre effect in the context of aesthetics and visual added value, the surfaces of the specimen were patterned in waves and pyramid patterns.

### 2.4. Dyeing Process

The dyeing procedure was carried out in Polycolor laboratory apparatus, Mathis AG, Switzerland, at OK 1:30, pH 4 was adjusted with 20% acetic acid, Kemika, Croatia, dye concentration (Dc) was 0.2, 0.5, 1, 2, 3 and 4% on the weight of the material (owm) or their combinations to obtain the ombre effect. To test the abrasion resistance, specimens were prepared with 3% Dc (owm). Dyeing in the Polycolor laboratory apparatus, Mathis ensures that all surfaces are available for dye adsorption, regardless the complexity of the specimen structure.

**Figure 2:**
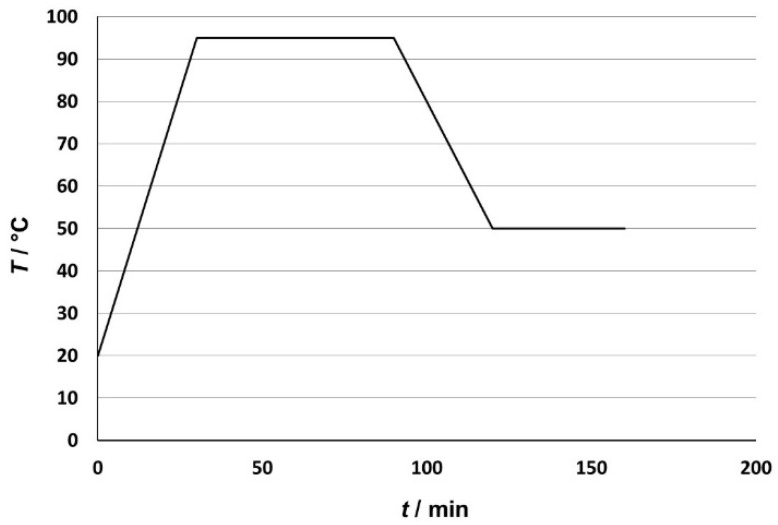
Schematic presentation of the dyeing process.

The dyeing process in the laboratory apparatus (Figure 2) starts with a gradual heating from 20 °C to 95 °C. This is followed by an isothermal process at 95 °C for 60 min, followed by cooling to 50 °C. The whole process took 160 min, including gradual heating and mandatory cooling. Due to the properties of ABS [7,8,9,10,11], it is important to follow the specified temperatures and time procedures, otherwise the 3D printed ABS specimens may be deformed.

Ombre dyeing was performed by capillary movement of the dye solution along the substrate. The substrate stands vertically in the dye solution, and the dye is applied vertically, giving the substrate a colored fade effect. By rotating the 3D printed ABS specimens, i.e., changing the direction and type of dye, different shaded effects were obtained.

### 2.5. Colorimetric Measurements

The evaluation of color characteristics of dyed 3D printed ABS specimens was objectively determined by spectrophotometric measurement with a spectrophotometer Spectra Flash 600 PLUS—CT, DataColor, Rotkreuz, Switzerland (with constant instrument aperture, D65, using d/8° geometry). The results are presented numerically according to the CIELAB system. The coordinates used to determine color values are L* for lightness, a* for redness (positive value) and greenness (negative value), b* for yellowness (positive value) and blueness (negative value), C* for chroma and h° for hue angle in the range of 0° to 360°. In addition, remission curves are presented, and the Kubelka-Munk coefficient (K/S) was calculated as a definition of color efficiency (color depth) in terms of K/S values, using the following Equation (1):

K/S = (1 − R)^2^/2R (1)

where K is the absorption coefficient, S is the scattering coefficient, and R is the reflectance of the dyed fabric.

Owing to statistical significance, the color measurements were made using the Datacolor ColorTools computer program and ‘‘Measuring until tolerance’’ command, which means that at least 10 measurements must be made, and the results are accepted only if the total color difference between each measurement is less than 0.1 (dE* < 0.1).

### 2.6. Color Abrasion Resistance

**Figure 3:**
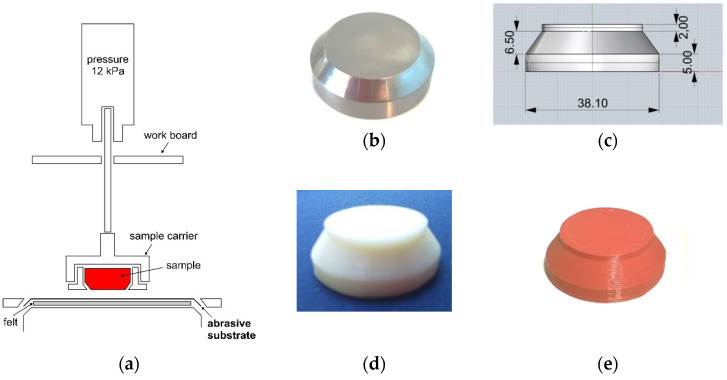
(**a**) Scheme of abrasion tester; (**b**) Original carrier cover; (**c**) CAD cover model; (**d**) example of a 3D printed ABS specimens prepared for the dyeing; (**e**) example of colored 3D printed ABS specimens.

Abrasion resistance was tested using the haberdashery Mesdan S.p.A. haberdashery, Puegnago Del Garda, Italy, model Martindale 2561E. The preparation of 3D printed ABS specimens was adapted for the abrasion resistance test as shown in Figure 3. The test was performed with a pressure of 12 kPa. The analysis was performed on (1). 3D printed ABS specimens produced by red filament dyed with the master batch and (2). specimens post dyed with C.I. Disperse Red 15 by exhaustion process.

The abrasives and their characteristics are defined in the Table 3.

The results are shown by weight loss after a number of cycles (250, 500, 750, 1000, 1250, 1500, 1750, 2000, 2250, 2500, 5000 and 10000 cycles) or until filling is showed. The mass loss (Δm) was determined based on the difference in mass after (m_a_) and before abrasion (m_0_) according to initial mass, Equation (2).

Δm (%) = ((m_a_ − m_0_)/m_0_)·100 (2)

For fastness properties analysis, the results are presented as total color difference values (dE_CIE76_) obtained by measuring and comparing the specimens before and after the fastness testing. Color difference values were calculated using Formula (3), defining the specimens before abrasion resistant tests as reference specimens.

dE_CIE76_ = ((dL*)^2^+(da*)^2^+(db*)^2^)^1/2^ (3)

where dL* is difference in lightness value, da* and db* are differences in a*/b* color coordinates indicating the change in L*a*b* color space position and indicating the change in chroma (C*) and hue (h°).

## 3. Results and Discussion

### 3.1. The Evaluation of Color Characteristics

**Figure 4:**
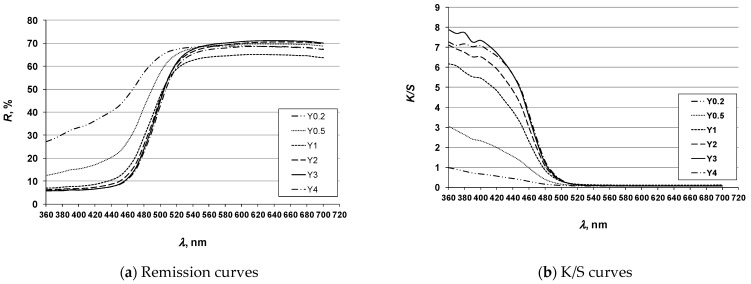
Spectral curves of yellow hue specimens (legend according to Table 4 and Table 5).

**Figure 5:**
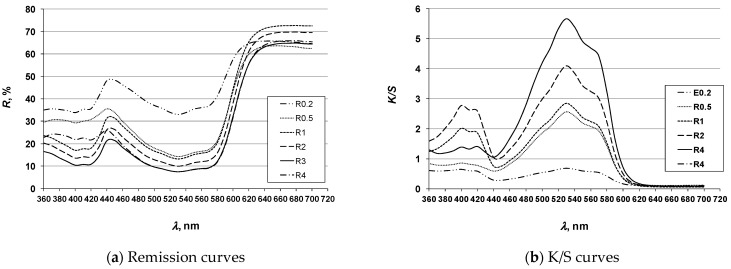
Spectral curves of red hue specimens (legend according Table 6 and Table 7).

**Figure 6:**
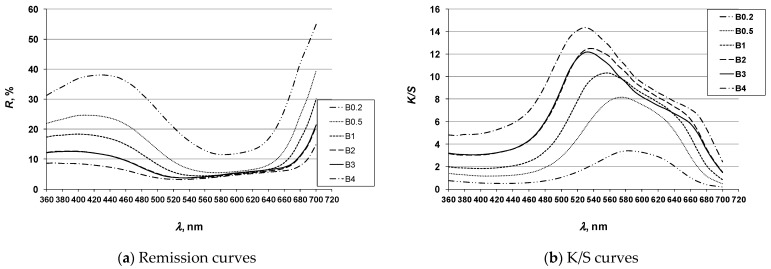
Spectral curves of blue hue specimens (legend according to Table 8 and Table 9).

To investigate the possibility of achieving multicolored effects, the 3D printed ABS specimens (Section 2.4) were dyed using the exhaust method with disperse dyes: Cibacet Yellow 2GC (Y), Cibacet Red 3B (R) and Foron Blue RD GLF (B) in a dye concentration gradient (Dc) of 0.2; 0.5; 1; 2; 3; and 4% owm. The dyed specimens are shown in Tables 4, 6 and 8. The results of spectrophotometric analysis of the specimens are presented by coloristic parameters according to the CIE system (Tables 5, 7 and 9), as well as by remission and K/S spectral curves (Figure 4, Figure 5 and Figure 6).

#### 3.1.1. Yellow Hue Specimens

The results of the analysis of the specimens of the yellow hue show a linear dependence of the chroma (C*) on the dye concentration, i.e., as the dye concentration increases, the value of the C* increases, reaching its constant value at a dye concentration of 2%, after which there is no further significant increase. The hue (h°) becomes more yellow with increasing concentration, ranging from yellow-green shades (h° = 106,13) for lower concentration towards more yellow shades for higher concentrations (h° = 95,74). The lightness (L*) decreases with increasing concentration, but the decrease is minimal and remains in the high lightness levels characteristic for the yellow hue (L* > 80). The graphically presented reflectance values define the spectral characteristic of the yellow-colored specimens. The uniformity of the absorption range (minimum curve) and the reflectance range (maximum curve) confirms that the spectral characteristic does not change with respect to dye concentration. Corresponding to the increase of chroma, the curve become more accented, meaning the distance between the absorption range and the reflectance range increase with increasing concentration. The K/S curves of the yellow-colored test specimens show a change in color depth with increasing concentration, and as expected, the most pronounced K/S curve with the highest K/S value was obtained for the dye concentration of 3% and 4%.

#### 3.1.2. Red Hue Specimens

For the red hue, the correlation between the dye concentration and the saturation parameter (C*) was the same as for the test specimens of the yellow hue, i.e., the increase in saturation with the increase in dye concentration. The range of the lightness value (L*) for the red hue obtained as a function of the dye concentration is larger compared to the yellow hue (from 70.81 to 48.22 for the dye concentration gradient from the lowest to the highest). At a dye concentration of 3%, there is a slight change in the spectral characteristics of the color, expressed by the change in the hue value (h°). The characteristic shape of the remission curve with two distinct reflection areas—in the blue and red spectrum—confirms the red-violet character of the obtained coloration, i.e., the characteristic magenta shade of the color. The uniform shape of the remission curve confirms the constancy of the hue. A slight shift towards a redder hue, visible in the change of the h° value for the dye concentration of 3%, is not observed in the remission. The differences in the heights of the remission curves confirms the range of lightness obtained, i.e., the decrease in the color lightness with the increase of the dye concentration. The K/S curves confirm the maximum color depth obtained for the specimens dyed with dye concentrations of 3% and 4%.

#### 3.1.3. Blue Hue Specimens

For blue specimen, the color characteristics show decreases in chroma (C*) as the color concentration increases, which is characteristic for the blue hue. The lightness value (L*) also decreases significantly with increasing dye concentration. A significant change in hue (h°) was obtained with increasing dye concentration. The hue changes from blue (h° = 276.19 for dye concentration 0.2%) to violet (h° = 314.17 for dye concentration 4%). The remission curves confirm the change in spectral characteristics of the color with a change in concentration. As the dye concentration increases, the pronounced reflectance peak in the blue spectrum decreases and the height approaches the maximum in the red spectrum. This confirms the change in spectral characteristics of color from blue to violet. The decrease in lightness with increasing dye concentration manifests itself in lower values of total reflectance. The K/S (color depth) curves confirm the above. The total value of K/S, i.e., the color depth, increases with increasing concentration.

### 3.2. Ombre Effect

The 3D printed objects ABS, dyed with a combination of disperse dyes, and the color effect obtained are shown in Table 10. The dilutions of the baths in terms of concentrations and gradients were adjusted based on the results shown in Section 3.1. In the test specimens shown in Table 10, a color effect of a gradual, fading color transition was obtained. From the point of view of artistic analysis, since the final application is the design of footwear, it can be said that the effects show a high degree of harmonic balance and the color combinations are in a positive contrast relationship.

### 3.3. Color Abrasion Resistance

To verify the functionalization of the prototypes, the abrasion resistance of the specimens was tested on six substrates with different textures: wool carpet (W), polypropylene carpets (PP), linoleum (L), and sandpaper with different grit sizes (SP 60, 80 and 240). This allows a simulation of the wear resistance test on indoor and outdoor substrates. The results were evaluated by determining the change in mass after the abrasion procedure. 3D printed test specimens made of ABS polymer, dyed with a masterbatch, and post dyed by the exhaust method were tested. Based on the previous results, the dye C.I. Disperse Red 15 with anthraquinone chromophore and a molecular weight of 239.23 g/mol was selected for the exhaust dyeing method.

**Figure 7:**
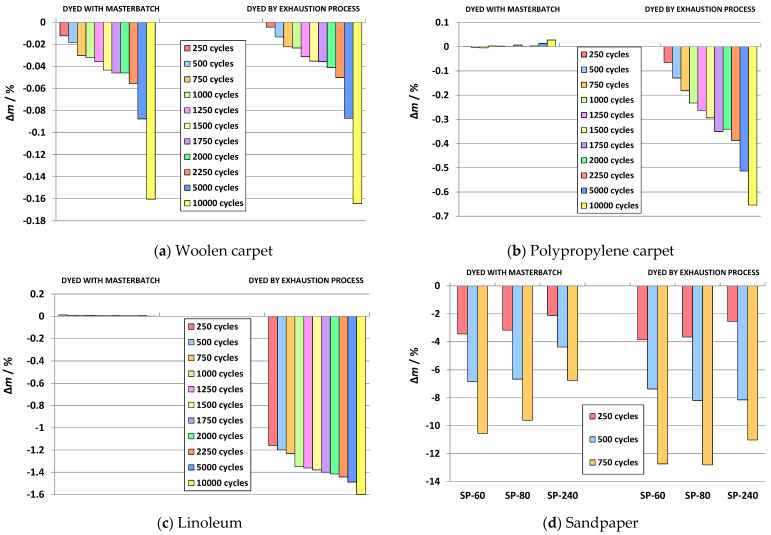
Weight loss of specimens on various abrasives.

In the case of the 3D printed ABS specimens dyed with a master batch, when comparing the results obtained regarding the abrasives, a high influence on the wear of the specimens is observed (Figure 7). This is especially confirmed by the results obtained for abrasives used as external substrates (sandpapers). For the same number of cycles (750), the change in weight loss ranges from 6.76 to 10.55%. In contrast, for abrasives used as indoor floor coverings, the weight loss is minimal at 10,000 cycles, from 0.02 to 0.16%. Based on the results of the abrasive resistance test obtained with the indoor floor coverings, it can be noticed that the test specimens dyed with a master batch have excellent wear resistance and their possible application for the manufacture of shoe heels would be justified. On the other hand, the results of the abrasive resistance test under outdoor conditions show that the outer layers of the test specimens were worn out with a significantly lower number of cycles (750), which is to be expected given the roughness of such solid substrates.

The influence of the structure of the abrasives is also observed for the post dyed specimens (Figure 7). Different types of abrasive paper show a change in weight loss from 11.03 to 12.81% for the same number of cycles (750). In contrast, for the abrasives used as indoor floor coverings, the changes in the form of weight loss and at 10,000 cycles were found to be minimal in relation to the initial weight, from 0.16 to 1.48%. The weight loss of the specimens is greatest for the sandpapers B-80, −12.81%, for B-60 it is −12.74 and for B-240 the weight loss is −11.03%. From Figure 7, it can be seen that the specimens post dyed with disperse dyes and abrased on carpet and linoleum substrates (T- PP, T-V and LIN) show higher weight loss than the specimens dyed with a master batch. The weight loss of the specimens abrased on the substrate T- PP is 0.65%, on the substrate T-V the weight loss is 0.16%, while for the specimens weard on the linoleum substrate after 10,000 cycles a weight loss of 1.48% was observed.

As for the printing parameters such as infill density and pattern as well as the interiour structure of the printed specimen, the results confirmed that they have no influence on the abrasion resistance.

### 3.4. Implementation

**Figure 8:**
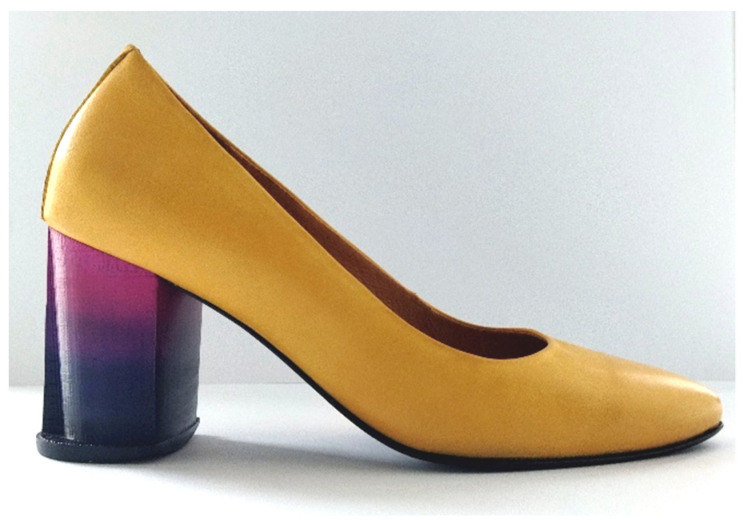
Realized prototype of women’s functional shoes.

The industrial realization of the performed scientific research was carried out in cooperation with the Croatian shoe factory Ivančica d.d. (Figure 8.). It was precisely the demand of the industry to influence the aesthetics of the heels.

**Figure 9:**
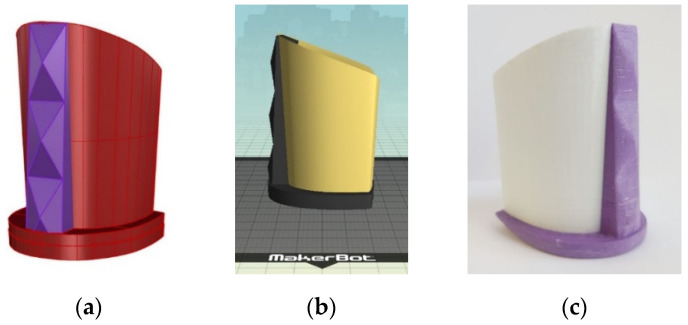
Making of the heel prototype: (**a**) CAD model; (**b**) positioning on the 3D printer working surface; (**c**) 3D printed ABS prototype.

The incorporation of a prototype of 3D printed and subsequently painted heels from ABS into a functional model of women’s shoes is combined with the classic production of shoes within the industrial footwear production with the aim of creating personalized models of wearable shoes or smaller limited series. Based on the scanned CAD model of women’s pumps height 75 mm No. 37 (factory name of the mold Pia) in the computer program Rhinoceros 5, a CAD model of the prototype heel was constructed (Figure 9a).

After verifying that the CAD model is correct, the STL files are transferred to the desktop 3D printer MakerBot Replicator 2X and the 3D printing parameters are set in MakerWare (printer software). The parameters of the 3D print were determined based on conducted research of mechanical properties (flexural and compressive properties and impact) of 3D printed test specimen in accordance with the targeted application to the final product, prototypes of shoe sole parts [24]. The printing parameters of the specimens are listed in Table 2 (Section 2.3). 

The vertical orientation of the heel prototype print was determined in accordance with the orientation of the final product [22] as well as the central positioning on the working surface of the printer (Figure 9b). Considering the selected parameters and the orientation of the print, the total estimated heel print material consumption is 86 g, and the time required for printing 8 h. The results of the 3D printed heel prototype on the MakerBot Replicator 2X desktop printer made of ABS in two colors (white and purple) are shown in Figure 9c.

The prototype made of ABS was dyed with a combination of C.I. Disperse Red 15 (DR15) and C.I. Disperse Blue 27 (DB27) by the exhoustion process. The achieved colored effect is shown in Table 11. The dilution of the color mix with respect to concentrations and gradients was adjusted based on the results shown in Section 3.1.

The upper of the realized prototype is a model of a classic women’s shoe, with an ergonomically modeled pattern, made of leather, which is still one of the most valued and high-quality materials in footwear construction. The process of installing the heel was carried out in an industrial way and with technologies for assembling the upper and lower footwear parts. All of this was preformed according to all the operations of the assembly phase. The prominence of the heel prototype, i.e., the application of new technologies (3D printing) in combination with traditional footwear making methods, is emphasized by the choice of complementary colors of the upper material in relation to the heel (Figure 8).

## 4. Conclusions

The results of this research can have a significant impact on the added value of 3D printed ABS products, as it has been shown that a color hue or even a variegated pattern with good abrasion resistance can be produced for prototypes. These effects are achieved by dyeing in the exhaust process with disperse dyes. Due to their chemical structure, they penetrate the structure of the material ABS, forming hydrogen bonds and providing satisfactory resistance. This is confirmed by the high values of the color depth for the 4% of dyes used, 8 for the yellow hue, K/S = 6 for the red hue and 14 for the blue hue.

Due to the chemical structure of ABS and the shape of the test specimens, disperse dyes do not penetrate deeply into the structure but bind the surface and after applying a dye concentration of more than 3%, the effect of increasing the concentration on the color depth is not observed. By comparing the properties of dyes with different chemical structure of chromophores and different relative molecular weights, the use of azo dyes is preferred over anthraquinones and dyes with lower relative molecular weights to achieve uniform color and good abrasion resistance.

It has been shown that there is a need to integrate methods for objective evaluation of color into the field of manufacturing works to ensure color reproducibility, quality assurance of color reproduction, and the esthetic aspect of objects to be integrated into design objects in the future. Looking at the results of the multicolor (“ombre”) effects, the opportunity to add value in the visual effects created by limiting printing to two colors in most desktop 3D printers can be recognized.

The results of the abrasive resistance test show that the test bodies dyed in the mass (original ABS dyed polymer) are on average about 10% more abrasive resistant compared to the specimens dyed afterwards with disperse dyes.

Considering the lack of scientific research on the process of post dyeing 3D printed ABS products with disperse dyes and the quality of the results, it can be concluded that this is an innovation that can significantly contribute to the development of 3D printed colored products.

## Figures and Tables

**Table 1:** Used disperse dyes.

Dyes	Label	Molecule Chemical Constitution
Cibacet Yellow 2GC, BASF, Switzerland; C.I. Disperse yellow 3	DY3 or Y	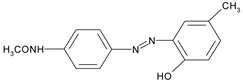
Cibacet Red 3B, BASF, Switzerland; C.I. Disperse Red 15	DR15 or R	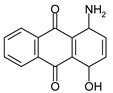
Foron Blue RD GLF, Sandoz, Switzerland; C.I. Disperse Blue 27	DB27 or B	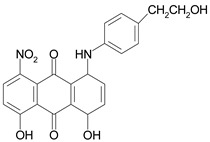

**Table 2:** The print parameters of the test specimens.

Print Parameter	Value
Layer thickness	0.15 mm
Infill density	40%
Number of shells	3
Infill build speed	90 mm/s
Shell speed	40 mm/s
Infill Pattern	linear 45°
Roof Thickness	1.00 mm
Temperature nozzle	205 °C

**Table 3:** Specification of abrasives.

Abrasive Substrates	Label	Thickness, mm
Woolen carpet	W	4.80
Polypropylene carpet	PP	1.52
Linoleum	L	1.28
Sandpaper, grit sizes 60	SP-60	0.94
Sandpaper, grit sizes 80	SP -80	0.74
Sandpaper, grit sizes 240	SP-240	0.50

**Table 4:** Specimens dyed with Disperse yellow 3 (DY3).

Specimens					
Y0.2	Y0.5	Y1	Y2	Y3	Y4
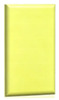	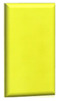	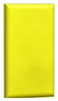	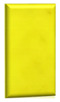	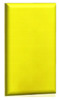	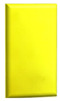

**Table 5:** Color parameters of Specimens dyed with dye Disperse yellow 3 (DY3).

Specimens	Dc/% (owm)	L*	a*	b*	C*	h°
Y0.2	0.2	85.29	−6.01	20.79	21.64	106.13
Y0.5	0.5	84.13	−8.38	43.2	44.01	100.98
Y1	1	80.79	−8.34	57.35	57.96	98.28
Y2	2	82.43	−7.05	65.53	65.91	96.14
Y3	3	82.66	−7.07	69.14	69.50	95.84
Y4	4	81.59	−6.87	68.34	68.68	95.74

**Table 6:** Specimens dyed with Disperse Red 3.

Specimens					
R0.2	R0.5	R1	R2	R3	R4
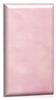	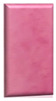	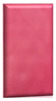	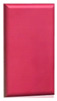	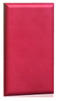	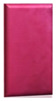

**Table 7:** Color parameters of specimens dyed with dye Disperse Red 15 (DR15).

Specimens	Dc/% (owm)	L*	a*	b*	C*	h°
R0.2	0.2	70.81	20.17	−2.54	20.33	352.84
R0.5	0.5	56.97	39.25	−8.56	40.17	347.70
R1	1	56.77	41.51	−2.12	41.57	357.08
R2	2	52.38	44.57	−1.34	44.59	358.28
R3	3	48.67	47.20	0.40	47.21	0.48
R4	4	48.22	48.81	−7.23	49.34	351.57

**Table 8:** Specimens dyed with Disperse blue 27 (DB27).

Specimens					
B0.2	B0.5	B1	B2	B3	B4
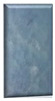	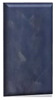	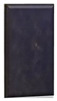	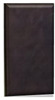	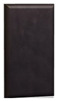	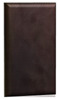

**Table 9:** Color parameters of specimens dyed with dye Disperse blue 27 (DB27).

Specimens	Dc/% (owm)	L*	a*	b*	C*	h°
B0.2	0.2	48.97	3.24	−29.92	30.10	276.19
B0.5	0.5	34.95	9.53	−32.29	33.67	286.44
B1	1	29.69	12.82	−27.87	30.67	294.70
B2	2	26.12	13.15	−19.86	23.82	303.51
B3	3	26.70	13.83	−18.74	23.29	306.43
B4	4	24.09	11.29	−11.63	16.21	314.17

**Table 10:** Specimens obtained with a mixture of dyes with an ombre effect (D_c_ according owm).

Specimens	Dyes	Dye Direction	Specimens	Dyes	Dye Direction	Specimens	Dyes	Dye Direction
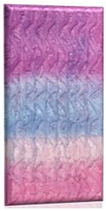	3% DR15 + 1% DB27	↓	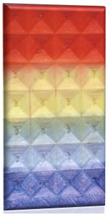	3% DR15 + 2% DY3	↓	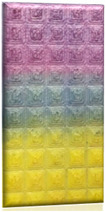	3% DR15 + 1% DB27	↓
	1% DB27	↓					1% DB27	↓
	3% DR15 + 1% DB27	↑		1% DB27	↑		3% DY3	↑
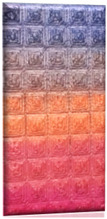	3% DB27	↓	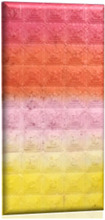	3% DR15	↓	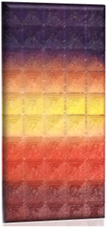	3% DR15 + 1% DY3 3% DB27	↓

	3% DR15 + 2% DY3	↑		3% DR15 + 3% DY3	↓		3% DY3	↑
	3% DR15	↑		1% DR15	↑		3% DR15 + 3% DY3	↑

**Table 11:** Heel prototype obtained with a mixture of dyes with an ombre effect.

Prototype Shoe Heel	Dyes	Dye Direction
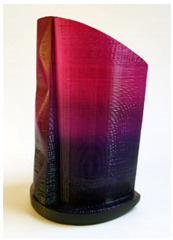	1% DB27 3% DR15 3% DR15 + 1% DB27 3% DR15	↓ ↓ ↓ ↑

## Data Availability

The data presented in this study are available on request from the corresponding author.

## Sample Availability

Samples of the compounds are available from the authors.
